# Poor health literacy associated with stronger perceived barriers to breast cancer screening and overestimated breast cancer risk

**DOI:** 10.3389/fonc.2022.1053698

**Published:** 2023-01-05

**Authors:** Paul K. M. Poon, King Wa Tam, Thomas Lam, Arthur K. C. Luk, Winnie C. W. Chu, Polly Cheung, Samuel Y. S. Wong, Joseph J. Y. Sung

**Affiliations:** ^1^ Jockey Club School of Public Health and Primary Care, The Chinese University of Hong Kong, Hong Kong, Hong Kong SAR, China; ^2^ Department of Imaging and Interventional Radiology, The Chinese University of Hong Kong, Hong Kong, Hong Kong SAR, China; ^3^ Hong Kong Breast Cancer Foundation, Hong Kong, Hong Kong SAR, China; ^4^ Lee Kong Chian School of Medicine, Nanyang Technological University, Singapore, Singapore

**Keywords:** health literacy, cancer screening (MeSH), barrier, risk perception, overestimate

## Abstract

**Background:**

Low health literacy (HL) is negatively associated with mammography screening uptake. However, evidence of the links between poor HL and low mammography screening participation is scarce.

**Methods:**

We conducted a cross-sectional questionnaire survey among participants of a cancer screening program. We measured HL using a validated Chinese instrument. We assessed breast cancer screening-related beliefs using the Health Belief Model and the accuracy of risk perception. We used multivariable regression models to estimate the relationship between HL and the outcomes.

**Results:**

A total of 821 females were included. 264 (32.2%) had excellent or sufficient, 353 (43.0%) had problematic, and 204 (24.8%) had inadequate health literacy (IHL). Women with IHL were more likely to agree that high price (β = -0.211, 95% CI -0.354 to -0.069), lack of time (β = -0.219, 95% CI -0.351 to -0.088), inconvenient service time (β = -0.291, 95% CI -0.421 to -0.160), long waiting time (β = -0.305, 95% CI -0.447 to -0.164), fear of positive results (β = -0.200, 95% CI -0.342 to -0.058), embarrassment (β = -0.225, 95% CI -0.364 to -0.086), fear of pain (β = -0.154, 95% CI -0.298 to -0.010), fear of radiation (β = -0.177, 95% CI -0.298 to -0.056), lack of knowledge on service location (β = -0.475, 95% CI -0.615 to -0.335), and lack of knowledge on mammography (β = -0.360, 95% CI -0.492 to -0.228) were barriers. They were also less likely to have an accurate breast cancer risk perception (aOR 0.572, 95% CI 0.341 to 0.956).

**Conclusion:**

Women with lower HL could have stronger perceived barriers to BC screening and an over-estimation of their breast cancer risk. Tackling emotional and knowledge barriers, financial and logistical assistance, and guidance on risk perception are needed to increase their breast cancer screening uptake.

## 1 Introduction

Breast cancer (BC) is the world’s most prevalent cancer among females with 2.26 million new cases and over 680,000 deaths in 2020 (1). BC screening is an important public health intervention to lessen the disease burden. Evidence showed that mammography screening could effectively reduce BC mortality (2, 3). Annual or biennial mammography screening has been widely adopted in cancer screening guidelines worldwide (4). However, the low uptake of BC screening remains a major concern; for instance, studies showed a screening rate of 32.1% in the United States (5) and 8-43% adherence to breast, colorectal and cervical cancer screening guidelines in Canada (6).

Having an adequate level of health literacy (HL) was shown in a recent meta-analysis to increase participation in BC screening (7). A study in the United States investigated HL and sociodemographic variables including ethnicity, language, education, smoking status, insurance, employment, income, and family history of BC. It found that, among all the factors considered, HL had the strongest association with adherence to mammography screening (8). Low HL was also shown to be negatively associated with up-to-date BC screening adhering to official guidelines (9). Indeed, the World Health Organization advocates empowering communities and improving HL as the first step for effective strategies for the promotion of early diagnosis (10). However, evidence on the links between poor HL and low BC screening participation is scarce. It is important to identify specific barriers or facilitators among people with poor HL to inform BC screening strategies catering for the needs of different people along the HL continuum.

On the other hand, most recommendations on BC screening are risk-based (4). Besides, evidence also showed that HL affected participation in non-recommended BC screening (11) which could be fuelled by an inaccurate risk perception. To further understand the association between BC screening behaviors and HL, investigating the role of perceived BC risk is of great importance. The association between HL and the perceived BC risk has not been widely researched and the available evidence is limited or inconclusive. For instance, a study in Ireland concluded that people with low HL tended to have an inaccurate perception of BC risk (12), while another study in Iran showed that HL level was not associated with perceived BC risk (13).

We hypothesized that women having a lower HL level would have more perceived barriers and less perceived facilitators for BC screening, and have less accurate BC risk perception.

## 2 Materials and methods

### 2.1 Study design and setting

This is a cross-sectional study including females who enrolled for mammography screening in the Multiple Cancer Screening Center (MCSC). This service is under a community-based multiple-cancer screening project, which was sponsored by the Hong Kong Jockey Club Charities Trust, a charitable organization, and run by the Faculty of Medicine of the Chinese University of Hong Kong. Further details of the project were described in a previous publication (14). Women registered online and were then contacted by trained staff by phone to confirm eligibility. Eligible individuals were females aged 50-75 years who did not have any of the following: a personal history of BC; swelling of all or part of the breast(s); breast skin irritation or dimpling; breast pain; nipple pain or the nipple turning inward; redness, scaliness or thickening of the nipple or breast skin; nipple discharge other than breast milk; lump(s) in the underarm area; or having received any BC screening test in the past 5 years. The screening service was free of charge.

Eligible women were invited to visit the MCSC to complete a structured self-administered questionnaire. Trained staff would provide on-site assistance if participants had difficulty understanding the questions. We measured HL using a validated Chinese instrument (HLS-SF12) (15). HLS-SF12 was derived from the 47-item European Health Literacy Questionnaire (HLS-EU-Q47) which was developed based on a comprehensive definition and a conceptual model of HL (16). The HLS-SF12 has been shown to retain the conceptual framework of HLS-EU-Q47 and have adequate psychometric properties including high reliability (Cronbach’s alpha = 0.85), good criterion-related validity and satisfactory item-scale convergent validity when used in different Asian countries (15). The components of HLS-SF12 include 12 health-related tasks representing the 12 dimensions of the conceptual model constructed from the four steps of information processing (finding health information, understanding health information, judging health information, and applying health information) (16). The women were asked to rate their perceived difficulty of each task on a 4-point Likert scale (1 = very difficult, 2 = difficult, 3 = easy, and 4 = very easy). The calculated HL scores ranged from 0 to 50 using the formula [(mean – 1) × (50/3)], where the mean was the mean of all the 12 items. The HL score of HLS-SF12 was shown to have a satisfactory correlation with the HL scores of HLS-EU-Q47 in multiple Asian countries, and the HLS-SF12 scores could explain 91-95% of the variance of the HL scores of HLS-EU-Q47 (15). Based on the HL scores, the HL levels were categorized as ‘inadequate’ (0–25), ‘problematic’ (>25–33), ‘sufficient’ (>33–42) and ‘excellent’ (>42–50) (17, 18). The ‘sufficient’ and ‘excellent’ levels were combined to a single level (>33–50) in the analysis to enhance statistical power. The required sample size was derived from the general rule of thumb for logistic regression by Bujang et al. (19) and calculated by the formula (n = 100 + 50i). With a total of 12 independent variables in our multivariable regression models, the recommended sample size was 700 (100 + 50*12).

### 2.2 Primary outcomes

The primary outcomes were BC screening-related beliefs or perceptions including BC risk perception. The 22 questions were developed based on the Health Belief Model and findings from previous studies on the health beliefs and behaviors of Chinese women on BC screening (20–22). The questions were then vetted by an expert panel consisting of public health specialists, family medicine doctors and experts in behavioral research. Several rounds of discussions were undertaken until a consensus was reached. To ensure clarity and comprehensibility, the questionnaire was pilot tested on 15 female MCSC participants, and face-to-face cognitive debriefings were conducted to verify that the translations of all the items on the questionnaire were understood in the same way by the target participants. Questions on perceived susceptibility to BC (1 question); perceived severity of BC (1 question); perceived benefits of BC screening (1 question); perceived barriers to BC screening (12 questions); and cues to action for undergoing BC screening (7 questions) were included. The women were asked to rate on a 4-point Likert scale (1 = strongly agree/very important, 2 = agree/important, 3 = disagree/unimportant, and 4 = strongly disagree/very unimportant) regarding the extent to which they agreed with the statements about their perceived susceptibility, perceived severity, perceived benefits, perceived importance of different barriers, and cues to action for BC screening. In the current study, Cronbach’s alpha was 0.8 for perceived barriers and 0.76 for cues to action, showing an acceptable level of internal reliability.

We also assessed the accuracy of BC risk perception based on the family history of BC. Family history is one of the strongest known risk factors for BC (23–25). According to the Hong Kong government recommendations on BC risk stratification of local females (26), women were classified as having an increased BC risk, as compared to the general public, if they have one first-degree female relative with BC diagnosed at ≤50 years of age; or two first-degree female relatives diagnosed with BC after the age of 50 years. The risk perception was regarded as concordant if a woman with increased risk answered “strongly agree” or “agree” to the statement “I have a very high chance of having breast cancer”; or a woman without an increased risk answered “disagree” or “strongly disagree”. Otherwise, the risk perception was regarded as discordant.

### 2.3 Covariates

Covariates included sociodemographic variables including age, place of birth, marital status, education level, personal and household income, and employment status. Data on self-rated health, history of common metabolic, gastrointestinal and pulmonary diseases including hypertension, diabetes, dyslipidemias, angina/ischaemic heart disease, stroke, fatty liver disease, chronic obstructive pulmonary diseases, gastroesophageal reflux disease, and history of any type of cancer (other than BC) were collected.

### 2.4 Statistical analyses

To test for any group differences across the three HL levels, the Chi-squared test was performed on categorical/dichotomous variables, and one-way ANOVA (analysis of variance) was performed on numerical variables. We used simple linear regression to estimate the relationship between HL and the primary outcomes. The dichotomous outcome of whether their BC risk perception was concordant with their family history was estimated using simple logistic regression. Further, multivariable linear and logistic regression models were used to adjust for potential confounders. The R software version 4.2.0 was used to perform the statistical analysis (27).

## 3 Results

A total of 821 females with a mean age near 58 years were included in the analysis. A total of 823 women who attended the mammography screening were recruited and 2 refused to join the study (response rate 99.8%). Over two-thirds were married or cohabitating and over half were employed. The mean HL level was 29.79 out of 50 with around one-third having excellent/sufficient HL and one-fourth having problematic HL. Education level and self-rated health were different among women with different HL levels. A minority (1.2%) reported a history of cancer (other than breast cancer) (**Table 1**).

**Table 1:** Characteristics of individuals by health literacy level.

	Level	Overall	Inadequate HL	Problematic HL	Sufficient/Excellent HL	p
**N**		821	204	353	264	
**Health literacy**	Mean score (SD)	29.79 (6.70)	21.31 (3.97)	29.39 (1.98)	36.89 (3.98)	<0.001
**Age**	Mean (SD)	57.96 (5.19)	59.36 (5.42)	57.60 (5.12)	57.35 (4.90)	<0.001
	50-54	265 (32.3)	46 (22.5)	122 (34.6)	97 (36.7)	0.01
	55-59	260 (31.7)	64 (31.4)	110 (31.2)	86 (32.6)	
	60-64	183 (22.3)	54 (26.5)	77 (21.8)	52 (19.7)	
	65+	113 (13.8)	40 (19.6)	44 (12.5)	29 (11.0)	
**Waist circumference**	Mean (SD)	90.46 (8.44)	91.63 (8.33)	90.18 (8.34)	89.95 (8.59)	0.07
**BMI**	Mean (SD)	25.99 (3.72)	26.17 (3.60)	26.03 (3.84)	25.79 (3.65)	0.53
**Education**	Primary school or below	113 (13.8)	57 (27.9)	40 (11.3)	16 (6.1)	<0.001
	Secondary 1-3	136 (16.6)	43 (21.1)	67 (19.0)	26 (9.8)	
	Secondary 4-7	370 (45.1)	74 (36.3)	173 (49.0)	123 (46.6)	
	College/ university or above	202 (24.6)	30 (14.7)	73 (20.7)	99 (37.5)	
**Marital status**	Married/ cohabitating	578 (70.4)	134 (65.7)	252 (71.4)	192 (72.7)	0.13
	Unmarried	112 (13.6)	28 (13.7)	49 (13.9)	35 (13.3)	
	Separated/ divorced	86 (10.5)	23 (11.3)	34 (9.6)	29 (11.0)	
	Widowed	45 (5.5)	19 (9.3)	18 (5.1)	8 (3.0)	
**Employment status**	Full-time	325 (40.0)	74 (37.2)	144 (40.8)	107 (41.2)	0.24
	Part-time	103 (12.7)	32 (16.1)	43 (12.2)	28 (10.8)	
	Retired	126 (15.5)	36 (18.1)	48 (13.6)	42 (16.2)	
	Housewife	210 (25.9)	45 (22.6)	98 (27.8)	67 (25.8)	
	Unemployed	29 (3.6)	10 (5.0)	13 (3.7)	6 (2.3)	
	Self-employed	19 (2.3)	2 (1.0)	7 (2.0)	10 (3.8)	
**Born in Hong Kong**	Yes	640 (78.0)	139 (68.1)	276 (78.2)	225 (85.2)	<0.001
	No	181 (22.0)	65 (31.9)	77 (21.8)	39 (14.8)	
**Personal income (HKD)**	5,000 or below	148 (21.1)	43 (25.1)	67 (22.0)	38 (16.9)	0.02
	5,001-10,000	120 (17.1)	38 (22.2)	50 (16.4)	32 (14.2)	
	10,001-15,000	137 (19.5)	40 (23.4)	54 (17.7)	43 (19.1)	
	15,001-20,000	98 (14.0)	20 (11.7)	45 (14.8)	33 (14.7)	
	20,001-30,000	89 (12.7)	14 (8.2)	44 (14.4)	31 (13.8)	
	30,001-40,000	50 (7.1)	8 (4.7)	23 (7.5)	19 (8.4)	
	40,000 or above	59 (8.4)	8 (4.7)	22 (7.2)	29 (12.9)	
**Household income (HKD)**	10,000 or below	66 (11.0)	22 (16.4)	29 (11.0)	15 (7.5)	<0.01
	10,001-20,000	144 (24.1)	41 (30.6)	66 (25.0)	37 (18.5)	
	20,001-30,000	121 (20.2)	24 (17.9)	55 (20.8)	42 (21.0)	
	30,001-40,000	105 (17.6)	24 (17.9)	47 (17.8)	34 (17.0)	
	40,001 or above	162 (27.1)	23 (17.2)	67 (25.4)	72 (36.0)	
**Self-reported health**	Excellent	23 (2.8)	5 (2.5)	6 (1.7)	12 (4.5)	<0.001
	Good	233 (28.4)	38 (18.6)	90 (25.5)	105 (39.8)	
	Fair	520 (63.3)	142 (69.6)	238 (67.4)	140 (53.0)	
	Poor	43 (5.2)	18 (8.8)	18 (5.1)	7 (2.7)	
	Very poor	2 (0.2)	1 (0.5)	1 (0.3)	0 (0.0)	
**Number of chronic conditions**	Mean (SD)	1.02 (1.14)	1.16 (1.23)	0.97 (1.12)	0.97 (1.08)	0.12
**Diabetes**	Yes	93 (11.3)	25 (12.3)	36 (10.2)	32 (12.1)	0.67
	No	728 (88.7)	179 (87.7)	317 (89.8)	232 (87.9)	
**Liver disease**	Yes	79 (9.6)	24 (11.8)	35 (9.9)	20 (7.6)	0.30
	No	742 (90.4)	180 (88.2)	318 (90.1)	244 (92.4)	
**Hypertension**	Yes	230 (28.0)	67 (32.8)	85 (24.1)	78 (29.5)	0.07
	No	591 (72.0)	137 (67.2)	268 (75.9)	186 (70.5)	
**Hyper- lipidemia**	Yes	186 (22.7)	53 (26.0)	82 (23.2)	51 (19.3)	0.22
	No	635 (77.3)	151 (74.0)	271 (76.8)	213 (80.7)	
**Ischemic heart disease**	Yes	6 (0.7)	2 (1.0)	3 (0.8)	1 (0.4)	0.71
	No	815 (99.3)	202 (99.0)	350 (99.2)	263 (99.6)	
**Chronic obstructive pulmonary disease**	Yes	8 (1.0)	1 (0.5)	5 (1.4)	2 (0.8)	0.51
	No	813 (99.0)	203 (99.5)	348 (98.6)	262 (99.2)	
**Stroke**	Yes	15 (1.8)	2 (1.0)	10 (2.8)	3 (1.1)	0.17
	No	806 (98.2)	202 (99.0)	343 (97.2)	261 (98.9)	
**Cirrhosis**	Yes	1 (0.1)	0 (0.0)	0 (0.0)	1 (0.4)	0.35
	No	820 (99.9)	204 (100.0)	353 (100.0)	263 (99.6)	
**Gastroesophageal reflux disease**	Yes	66 (8.0)	24 (11.8)	28 (7.9)	14 (5.3)	0.04
	No	755 (92.0)	180 (88.2)	325 (92.1)	250 (94.7)	
**Other co-morbidities**	Yes	142 (17.3)	38 (18.6)	53 (15.0)	51 (19.3)	0.32
	No	679 (82.7)	166 (81.4)	300 (85.0)	213 (80.7)	
**Cancer** **(any type other than breast cancer)**	Yes	10 (1.2)	1 (0.5)	5 (1.4)	4 (1.5)	0.55
	No	811 (98.8)	203 (99.5)	348 (98.6)	260 (98.5)	
**Family history of breast cancer**	Yes	55 (6.7)	16 (7.8)	20 (5.7)	19 (7.2)	0.57
	No	766 (93.3)	188 (92.2)	333 (94.3)	245 (92.8)	

HL, health literacy; SD, standard deviation; N, the number of observations. The p-values indicate the level of significance of chi-squared tests on categorical/dichotomous variables, and that of one-way ANOVA on numerical variables. Percentages (or standard deviation where specified) are in parenthesis.

Simple linear regression showed that perceived susceptibility and perceived severity of BC were higher in women with a lower HL level. Multiple perceived barriers to BC screening were stronger in women with lower HL levels. Perceptions of cues to action for undergoing BC screening were different by HL levels. Women with IHL were less likely to have a concordant BC risk perception (**Table 2**).

**Table 2:** Associations between screening-related perceptions and health literary (N=821).

Reference level: Sufficient/Excellent HL		Inadequate HL	Problematic HL
Type	Outcome	Coefficient	Coefficient
**Perceived susceptibility**			
	“I have a very high chance of having breast cancer”	-0.283*** (-0.389, -0.177)	-0.096* (-0.189, -0.003)
**Perceived severity**			
	“I will die in 1-2 years if I have breast cancer”	-0.247*** (-0.346, -0.147)	-0.045 (-0.132, 0.042)
**Perceived benefit**			
	“Mammography can detect breast cancer that I am not aware of.”	0.086 (-0.010, 0.182)	0.045 (-0.039, 0.129)
**Financial barrier**			
	“High price”	-0.192** (-0.313, -0.071)	-0.055 (-0.161, 0.050)
**Logistical barriers**			
	“Lack of time to do breast cancer screening”	-0.149** (-0.261, -0.038)	-0.147** (-0.244, -0.050)
	“Inconvenient service time”	-0.224*** (-0.334, -0.114)	-0.111* (-0.207, -0.015)
	“Long waiting time”	-0.299*** (-0.421, -0.177)	-0.135* (-0.242, -0.029)
**Emotional barriers**			
	“Fear of positive result”	-0.193** (-0.314, -0.073)	-0.139** (-0.244, -0.033)
	“Embarrassment”	-0.194** (-0.314, -0.074)	-0.083 (-0.187, 0.022)
	“Fear of pain”	-0.114 (-0.238, 0.011)	-0.027 (-0.135, 0.082)
	“Fear of radiation”	-0.136** (-0.240, -0.033)	0.017 (-0.073, 0.108)
**Knowledge barriers**			
	“No need to screen because of good health”	0.027 (-0.086, 0.140)	0.041 (-0.057, 0.139)
	“No recommendation from my doctor”	0.078 (-0.044, 0.199)	0.060 (-0.046, 0.166)
	“Lack of knowledge on service location”	-0.504*** (-0.625, -0.383)	-0.238*** (-0.344, -0.132)
	“Lack of knowledge on mammography”	-0.392*** (-0.505, -0.279)	-0.155** (-0.253, -0.056)
**Cues to action**			
	“One-stop multiple cancer screening service”	0.040 (-0.060, 0.139)	0.038 (-0.049, 0.125)
	“Fear of having breast cancer”	-0.176** (-0.281, -0.071)	-0.060 (-0.151, 0.032)
	“Healthcare professional recommendation”	-0.146** (-0.241, -0.050)	0.017 (-0.066, 0.100)
	“Relative/friend recommendation”	-0.055 (-0.151, 0.041)	0.000 (-0.084, 0.084)
	“Media information”	0.018 (-0.086, 0.122)	0.159*** (0.068, 0.249)
	“Free-of-charge service”	-0.051 (-0.159, 0.056)	0.055 (-0.039, 0.149)
	“Benefits of breast cancer screening”	0.013 (-0.083, 0.110)	0.073 (-0.011, 0.158)
**Risk concordance**			
	Concordant breast cancer risk perception	0.409*** (0.267, 0.622)	0.921 (0.612, 1.379)

HL; health literacy. 95% confidence intervals are in parenthesis. * p < 0.05; ** p < 0.01; *** p < 0.001. Simple linear regression was used to estimate the coefficients, except for risk concordance whose coefficients are odds ratios estimated by simple logistic regression.

Multivariable linear regression showed that, compared to excellent and sufficient HL, women with IHL were more likely to have higher perceived susceptibility and higher perceived severity of BC. They were more likely to agree that high price, a lack of time, inconvenient service time, long waiting time, a fear of positive results, embarrassment, a fear of pain, a fear of radiation, a lack of knowledge on service location, and a lack of knowledge on mammography were barriers to BC screening. Compared to excellent and sufficient HL, women with PHL were more likely to agree that a lack of time, inconvenient service time, long waiting time, a fear of positive results, a lack of knowledge on service location, and a lack of knowledge on mammography were barriers to BC screening. Women with IHL did not show a statistically significant difference in terms of perception of cues to action compared to those with excellent and sufficient HL, but women with PHL were less likely to agree that media information was an important cue to action. Regarding cue to action, compared to college/university or above education level, women with lower education level were more likely to agree that recommendations from healthcare professionals or friends/relatives or media information were important cues to action. Women with IHL were less likely to have a concordant BC risk perception (aOR 0.572). Lower likelihoods of concordant BC risk perception were also seen in women with positive family history of BC (aOR 0.302) and lower education level (lower secondary education aOR 0.372, primary school or below aOR 0.291) (**Table 3** is an abridged table, please refer to the **Supplementary Table S1** for the full results). Among women participating in BC screening, education level was the strongest determinant among all covariates on HL level (**Table S2**).

**Table 3:** Associations between screening-related perceptions and health literacy adjusted for covariates^#^ (N=701).

		Health literacy level		Family history of breast cancer
		Ref: Sufficient/Excellent		
		Inadequate	Problematic	
**Type**	Outcome	Coef.	Coef.	Coef.
**Perceived susceptibility**				
	“I have a very high chance of having breast cancer”	-0.164** (-0.285, -0.044)	-0.060 (-0.160, 0.040)	-0.454*** (-0.623, -0.284)
**Perceived severity**				
	“I will die in 1-2 years if I have breast cancer”	-0.200*** (-0.317, -0.083)	-0.033 (-0.130, 0.064)	0.061 (-0.103, 0.225)
**Perceived benefit**				
	“Mammography can detect breast cancer that I am not aware of.”	0.048 (-0.064, 0.160)	0.023 (-0.069, 0.116)	-0.055 (-0.213, 0.102)
**Financial barrier**				
	“High price”	-0.211** (-0.354, -0.069)	-0.074 (-0.193, 0.044)	0.069 (-0.131, 0.269)
**Logistical barriers**				
	“Lack of time to do breast cancer screening”	-0.219** (-0.351, -0.088)	-0.195*** (-0.304, -0.086)	-0.132 (-0.317, 0.053)
	“Inconvenient service time”	-0.291*** (-0.421, -0.160)	-0.136* (-0.244, -0.028)	-0.128 (-0.311, 0.055)
	“Long waiting time”	-0.305*** (-0.447, -0.164)	-0.165** (-0.282, -0.048)	-0.144 (-0.342, 0.055)
**Emotional barriers**				
	“Fear of positive result”	-0.200** (-0.342, -0.058)	-0.152* (-0.269, -0.034)	-0.231* (-0.430, -0.032)
	“Embarrassment”	-0.225** (-0.364, -0.086)	-0.067 (-0.182, 0.048)	-0.144 (-0.338, 0.051)
	“Fear of pain”	-0.154* (-0.298, -0.010)	0.004 (-0.115, 0.123)	-0.072 (-0.274, 0.130)
	“Fear of radiation”	-0.177** (-0.298, -0.056)	0.020 (-0.080, 0.120)	-0.099 (-0.269, 0.071)
**Knowledge barriers**				
	“No need to screen because of good health”	-0.017 (-0.151, 0.116)	0.036 (-0.075, 0.146)	-0.081 (-0.269, 0.106)
	“No recommendation from my doctor”	-0.027 (-0.169, 0.115)	0.045 (-0.073, 0.163)	0.055 (-0.145, 0.254)
	“Lack of knowledge on service location”	-0.475*** (-0.615, -0.335)	-0.206*** (-0.322, -0.090)	0.097 (-0.099, 0.294)
	“Lack of knowledge on mammography”	-0.360*** (-0.492, -0.228)	-0.113* (-0.222, -0.003)	-0.026 (-0.211, 0.160)
**Cues to action**				
	“One-stop multiple cancer screening service”	0.053 (-0.064, 0.170)	0.035 (-0.062, 0.132)	-0.036 (-0.200, 0.128)
	“Fear of having breast cancer”	-0.079 (-0.200, 0.041)	-0.014 (-0.114, 0.086)	0.051 (-0.118, 0.221)
	“Healthcare professional recommendation”	-0.059 (-0.172, 0.053)	0.065 (-0.029, 0.158)	0.151 (-0.008, 0.309)
	“Relative/friend recommendation”	0.072 (-0.042, 0.185)	0.037 (-0.056, 0.131)	-0.001 (-0.160, 0.158)
	“Media information”	0.113 (-0.010, 0.236)	0.231*** (0.129, 0.332)	0.170 (-0.003, 0.343)
	“Free-of-charge service”	-0.088 (-0.212, 0.037)	0.037 (-0.066, 0.141)	0.056 (-0.119, 0.232)
	“Benefits of breast cancer screening”	0.017 (-0.097, 0.131)	0.088 (-0.006, 0.183)	0.049 (-0.111, 0.209)
**Type**	Outcome	OR	OR	OR
**Risk concordance**				
	Concordant cancer screening risk perception	0.572* (0.341, 0.956)	1.034 (0.648, 1.640)	0.302*** (0.157, 0.584)

Coef.; coefficients. OR; odds ratio. 95% confidence intervals are in parenthesis. #Adjusted for age, number of chronic diseases, history of other cancers, family history of breast cancer, waist circumference, body mass index, education level, marital status, employment status, birthplace, and household income. * p < 0.05; ** p < 0.01; *** p < 0.001. Multiple linear regression was used to estimate the coefficients, except for risk concordance whose coefficients are odds ratios estimated by logistic regression. Each row represents a separate regression model. 120 were excluded from the model due to missing data on household income (N=120) and employment status (N=9). # Please refer to the **Supplementary Table S1** for the full results.

## 4 Discussions

In our study, over two-thirds of the female participants had PHL or IHL (**Table 1**). The proportion is high when compared to the 47% found in a study using the HLS-EU-Q47 scale in the European region (17). Regarding perceived barriers to BC screening, women with IHL held a stronger belief than those with excellent or sufficient HL that financial (high price), logistical (time constraint, inconvenient service time, long waiting time), emotional (fear of positive results, fear of radiation, embarrassment) and knowledge (lack of knowledge on service location and mammography) factors were barriers to BC screening (**Table 3**). Women with PHL also had a stronger belief that the lack of knowledge on mammography and fear of positive results were barriers to BC screening. These findings are consistent with a study in the United States, which showed that women with lower HL reported more emotional and knowledge barriers to BC screening (28). However, the same study also indicated that these women reported fewer logistical barriers, which is not consistent with our findings. This inconsistency could be multifactorial including cultural differences (29), differences in access to health care (30), or socioeconomic status (31), that would require further research to investigate the effects of these factors on the relationship between HL and BC screening. Nevertheless, our results showed that women with low HL would perceive stronger barriers to BC screening in several dimensions, and provided evidence of the links between low HL and low BC screening participation. Unlike barriers, we found that cues to action or facilitators for BC screening were less affected by HL levels. Apart from women with PHL who accorded lower importance to “media information”, we did not see statistically significant differences across the HL continuum in terms of the importance of BC screening facilitators (**Table 3**). Intriguingly, independent of HL level, women with different education levels apparently would accord different importance to facilitators like recommendations from healthcare professionals, friends/relatives, and media information on screening. It may warrant further studies to explore the differential effects of HL and education level on cues to BC screening.

Various HL-based interventions have been developed aiming to improve BC screening uptake in people with low HL. These interventions mainly focus on building HL skills (32) or providing educational materials (33). However, studies have shown that materials or counselling techniques adopted in these interventions might not be responsive to the needs of the recipients (34, 35). Our study helps inform the development of such interventions that can tackle the stronger emotional and knowledge barriers to BC screening among people with lower HL. In addition to education and empowerment, our results indicated that addressing external factors such as price, service hours and capacity are also important in reducing barriers to BC screening for people with low HL.

Moreover, women with lower HL in our study had poorer self-rated health than those with higher HL regardless of the number of chronic illnesses that they had (**Table 1**). This finding is consistent with a previous study among Chinese adults showing higher HL was positively associated with better self-rated health (36). Our subjects with PHL or IHL also agreed more strongly with a high own BC risk and a high severity of BC than women with excellent or sufficient HL (**Table 3**). Similar findings of higher perceived BC risk among women with low HL were also seen in another study (37). Furthermore, we also found an association between low HL and inaccuracy of BC risk perception. Compared to women with excellent or sufficient HL, those with IHL had a nearly two-fold increase in the odds of having BC risk perception discordant with their BC family history (**Table 3**). Since most national and international recommendations on BC screening are risk-based (4, 26), a shared and informed decision on BC screening should ideally be made by a woman after a discussion with her healthcare provider on her own risk level. Family history of BC is an important risk indicator (26) and is not rare (6.7% among our subjects, **Table 1**). Besides, our results also showed that women with a positive family history were more likely to have a higher perceived susceptibility to BC that were less likely to be accurate (**Table 3**). It indicates that guidance for these women is needed for a correct interpretation of their positive family history. Decision aids have been developed to assist women to come up with a more accurate risk perception (38). An overestimation of risk could lead to over-utilization of mammography screening or other healthcare services as shown in a study in the United States (39). This could be a possible link to the observed suboptimal including overutilization of healthcare resources by people with low HL (40). Age-based screening recommendations are widely adopted internationally (4) that women aged 50 or above are recommended for regular mammography screening. While this is a risk-based and pragmatic approach for a public health policy, our results implied that women with low HL would require more guidance on BC risk perception. Besides screening decisions, correcting an overestimation of risk would reduce the associated unnecessary worries and psychological distress (41, 42), which could be equally important to an individual’s well-being.

### Limitations

First, the cross-sectional design of this study could not directly infer a causal relationship between HL levels and BC screening-related beliefs. A longitudinal study would provide further insights. Second, only mammography screening was assessed. That said, mammography is the most widely adopted BC screening method in population-based BC screening (4, 26). Third, we studied participants of a cancer screening program who could be more health conscious and might have a higher HL than the general population. We might not be able to assess if there was an over-representation of women with higher HL in our sample as data on the overall HL picture of the Hong Kong general population were not available. Nevertheless, the percentage of recruited subjects from the three regions of Hong Kong was 13.5%, 26.8%, 58.8% and 1.3% for Hong Kong Island, Kowloon, and New Territories and Islands respectively, that closely resembled the data from Hong Kong population census on population distribution (43). Moreover, this study did not aim to provide an estimate of the general HL level of the local population but aimed to investigate associations between HL and BC screening-related beliefs. The possible under-representation of people with low HL in our sample might affect the power of our study but should not have a marked impact on the direction of associations. Fourth, all subjects had already participated in BC screening in this study that did not provide a comparison unscreened group for further analysis (e.g. mediation analysis) of the mechanism among HL, screening beliefs and risk perception, and screening uptake. Further studies including both screened and unscreened subjects are needed to investigate the mechanism. Nevertheless, even only among screening participants, our study results supported the hypothesis that women with low HL would have more perceived barriers to BC screening and a less accurate BC risk perception.

## 5 Conclusion

Compared to women with excellent or sufficient HL, women with lower HL could have stronger perceived barriers to BC screening on multiple aspects including financial, logistical, emotional, and knowledge barriers. They also had an overestimation of their own BC risk. Besides addressing emotional and knowledge barriers in BC screening promotion strategies, providing financial and logistical assistance is also needed to increase BC screening uptake for women with low HL. They also require guidance on BC risk perception.

## Data availability statement

The raw data supporting the conclusions of this article will be made available by the authors, without undue reservation.

## Ethics statement

This study was approved by the Joint Chinese University of Hong Kong – New Territories East Cluster Clinical Research Ethics Committee (CRE-2018.165). The patients/participants provided their written informed consent to participate in this study.

## Author contributions

Conceptualization, investigation, methodology by PP, KT, AL, JS, and SW; data curation and analysis by PP and KT; funding acquisition by JS; writing - original draft by PP, KT, and SW; writing - review and editing by TL, AL, WC, PC, and JS; supervision by SW and JS. All authors contributed to the article and approved the submitted version.
