# Supplementary material for: Poor health literacy associated with stronger perceived barriers to breast cancer screening and overestimated breast cancer risk
Source: Front Oncol. 2023 Jan 5;12:1053698. doi: 10.3389/fonc.2022.1053698 (PMC9850080; doi:10.3389/fonc.2022.1053698)
Supplement: Supplementary file 1 [file DataSheet_1.docx]

Table S1. Associations between screening-related perceptions and health literacy adjusted for covariates, full results (N=701)

|  |  | | Health literacy level | | | Age | | | Number of chronic diseases | History of other cancers | Family history of breast cancer | | Waist circumfer-ence | BMI | Education | | | Marital status | | | | Employment status | | | | | Born in Hong Kong | | Household income | | | |
| --- | --- | --- | --- | --- | --- | --- | --- | --- | --- | --- | --- | --- | --- | --- | --- | --- | --- | --- | --- | --- | --- | --- | --- | --- | --- | --- | --- | --- | --- | --- | --- | --- |
|  |  | | Ref: Sufficient/Excellent | | | Ref: 50-54 | | |  |  |  | |  |  | Ref: College/university | | | Ref: Married/cohabitating | | | | Ref: Full-time | | | | |  | | Ref: 10,000 or below | | | |
|  |  | | Inadequate | | Problematic | 55-59 | 60-64 | 65+ |  |  |  |  | |  | Primary school | Secondary school 1-3 | Secondary school 4-7 | Unmarried | Separated  /divorced | Widowed | Part-time | | Retired | Housewife | Unemployed | Self-employed |  | 10,001-20,000 | | 20,001-30,000 | 30,000+ |  |
| Type | Outcome | | Coef. | | Coef. | Coef. | Coef. | Coef. | Coef. | Coef. | Coef. | Coef. | | Coef. | Coef. | Coef. | Coef. | Coef. | Coef. | Coef. | Coef. | | Coef. | Coef. | Coef. | Coef. | Coef. | Coef. | | Coef. | Coef. |  |
| Perceived susceptibility | |  | |  | |  |  |  |  |  |  |  | |  |  |  |  |  |  |  |  | |  |  |  |  |  |  | |  |  |  |
|  | “I have a very high chance of having BC” | | -0.164** (-0.285, -0.044) | | -0.060 (-0.160, 0.040) | -0.052 (-0.160, 0.055) | 0.088 (-0.051, 0.227) | 0.063 (-0.108, 0.233) | -0.016 (-0.054, 0.022) | -0.374 (-0.776, 0.028) | -0.454*** (-0.623, -0.284) | -0.004 (-0.013, 0.004) | | 0.011 (-0.007, 0.030) | -0.388*** (-0.565, -0.212) | -0.229** (-0.387, -0.071) | -0.111 (-0.228, 0.006) | -0.063 (-0.198, 0.073) | 0.164* (0.025, 0.302) | 0.121 (-0.067, 0.308) | 0.068 (-0.082, 0.219) | | 0.026 (-0.141, 0.194) | 0.071 (-0.070, 0.212) | -0.075 (-0.357, 0.207) | -0.049 (-0.324, 0.225) | 0.015 (-0.093, 0.124) | 0.018 (-0.104, 0.139) | | 0.239** (0.072, 0.406) | 0.096 (-0.071, 0.263) |  |
| Perceived severity | | |  | |  |  |  |  |  |  |  |  | |  |  |  |  |  |  |  |  | |  |  |  |  |  |  | |  |  |  |
|  | “I will die in 1-2 years if I have BC” | | -0.200*** (-0.317, -0.083) | | -0.033 (-0.130, 0.064) | -0.019 (-0.124, 0.085) | 0.012 (-0.123, 0.147) | -0.050 (-0.215, 0.115) | -0.010 (-0.047, 0.027) | -0.097 (-0.487, 0.292) | 0.061 (-0.103, 0.225) | -0.007 (-0.015, 0.001) | | 0.002 (-0.017, 0.020) | -0.354*** (-0.525, -0.184) | -0.146 (-0.299, 0.008) | -0.102 (-0.215, 0.012) | 0.028 (-0.103, 0.159) | 0.150* (0.016, 0.285) | 0.147 (-0.034, 0.329) | 0.017 (-0.129, 0.163) | | -0.042 (-0.204, 0.121) | -0.090 (-0.227, 0.047) | 0.078 (-0.195, 0.352) | -0.217 (-0.483, 0.049) | -0.010 (-0.115, 0.095) | 0.023 (-0.095, 0.140) | | 0.138 (-0.024, 0.300) | 0.027 (-0.135, 0.189) |  |
| Perceived benefit | | |  | |  |  |  |  |  |  |  |  | |  |  |  |  |  |  |  |  | |  |  |  |  |  |  | |  |  |  |
|  | “MMG can detect BC that I am not aware of.” | | 0.048 (-0.064, 0.160) | | 0.023 (-0.069, 0.116) | -0.027 (-0.127, 0.073) | -0.130* (-0.260, -0.001) | -0.238** (-0.397, -0.080) | 0.016 (-0.019, 0.052) | -0.192 (-0.565, 0.182) | -0.055 (-0.213, 0.102) | 0.001 (-0.006, 0.009) | | 0.005 (-0.012, 0.023) | 0.150 (-0.013, 0.314) | 0.145 (-0.002, 0.292) | 0.093 (-0.016, 0.201) | 0.079 (-0.047, 0.205) | -0.085 (-0.214, 0.043) | 0.039 (-0.135, 0.213) | 0.089 (-0.051, 0.229) | | 0.136 (-0.019, 0.292) | 0.025 (-0.106, 0.156) | -0.183 (-0.446, 0.079) | -0.243 (-0.498, 0.013) | -0.007 (-0.108, 0.094) | -0.024 (-0.137, 0.089) | | 0.036 (-0.119, 0.191) | 0.009 (-0.146, 0.164) |  |
| Financial barrier | | |  | |  |  |  |  |  |  |  |  | |  |  |  |  |  |  |  |  | |  |  |  |  |  |  | |  |  |  |
|  | “High price” | | -0.211** (-0.354, -0.069) | | -0.074 (-0.193, 0.044) | 0.054 (-0.074, 0.181) | 0.075 (-0.089, 0.240) | 0.074 (-0.128, 0.275) | -0.020 (-0.065, 0.024) | 0.094 (-0.381, 0.570) | 0.069 (-0.131, 0.269) | -0.009 (-0.019, 0.001) | | 0.011 (-0.011, 0.034) | 0.045 (-0.163, 0.253) | -0.072 (-0.259, 0.115) | -0.049 (-0.187, 0.089) | -0.137 (-0.297, 0.023) | -0.050 (-0.214, 0.114) | -0.045 (-0.266, 0.176) | 0.127 (-0.051, 0.305) | | 0.183 (-0.015, 0.381) | 0.055 (-0.111, 0.222) | 0.241 (-0.093, 0.574) | 0.131 (-0.193, 0.456) | -0.077 (-0.205, 0.051) | 0.006 (-0.138, 0.149) | | 0.183 (-0.014, 0.381) | 0.232* (0.034, 0.429) |  |
| Logistical barriers | | |  | |  |  |  |  |  |  |  |  | |  |  |  |  |  |  |  |  | |  |  |  |  |  |  | |  |  |  |
|  | “Lack of time to do BC screening” | | -0.219** (-0.351, -0.088) | | -0.195*** (-0.304, -0.086) | 0.187** (0.069, 0.304) | 0.203** (0.051, 0.355) | 0.177 (-0.009, 0.363) | -0.011 (-0.052, 0.031) | -0.062 (-0.501, 0.377) | -0.132 (-0.317, 0.053) | -0.006 (-0.015, 0.003) | | 0.006 (-0.015, 0.026) | 0.040 (-0.152, 0.233) | 0.116 (-0.057, 0.288) | 0.109 (-0.019, 0.237) | -0.013 (-0.161, 0.135) | 0.058 (-0.093, 0.210) | 0.190 (-0.014, 0.395) | 0.164 (0.000, 0.329) | | 0.223* (0.040, 0.406) | 0.076 (-0.078, 0.230) | 0.194 (-0.114, 0.502) | 0.068 (-0.232, 0.368) | 0.012 (-0.107, 0.130) | 0.085 (-0.047, 0.218) | | 0.073 (-0.110, 0.255) | -0.003 (-0.185, 0.180) |  |
|  | “Inconvenient service time” | | -0.291*** (-0.421, -0.160) | | -0.136* (-0.244, -0.028) | 0.157** (0.041, 0.274) | 0.210** (0.060, 0.361) | 0.217* (0.033, 0.401) | -0.013 (-0.054, 0.028) | 0.000 (-0.433, 0.434) | -0.128 (-0.311, 0.055) | -0.001 (-0.010, 0.007) | | -0.005 (-0.025, 0.015) | 0.002 (-0.188, 0.191) | 0.073 (-0.098, 0.243) | 0.077 (-0.050, 0.203) | 0.010 (-0.136, 0.156) | 0.026 (-0.124, 0.176) | 0.203* (0.002, 0.405) | 0.198* (0.035, 0.360) | | 0.248** (0.068, 0.429) | 0.164* (0.011, 0.316) | 0.107 (-0.197, 0.412) | 0.208 (-0.089, 0.504) | 0.010 (-0.107, 0.128) | 0.093 (-0.038, 0.223) | | 0.148 (-0.033, 0.328) | 0.074 (-0.106, 0.255) |  |
|  | “Long waiting time” | | -0.305*** (-0.447, -0.164) | | -0.165** (-0.282, -0.048) | 0.181** (0.055, 0.308) | 0.065 (-0.099, 0.228) | 0.217* (0.017, 0.417) | -0.018 (-0.063, 0.026) | -0.009 (-0.480, 0.462) | -0.144 (-0.342, 0.055) | 0.001 (-0.009, 0.011) | | -0.012 (-0.034, 0.010) | 0.007 (-0.200, 0.213) | -0.001 (-0.187, 0.185) | 0.038 (-0.099, 0.175) | 0.001 (-0.158, 0.160) | -0.048 (-0.211, 0.115) | -0.070 (-0.289, 0.150) | 0.128 (-0.049, 0.304) | | 0.150 (-0.046, 0.346) | 0.028 (-0.138, 0.193) | 0.278 (-0.053, 0.609) | 0.156 (-0.166, 0.478) | -0.121 (-0.249, 0.006) | -0.007 (-0.149, 0.136) | | 0.220* (0.025, 0.416) | 0.261** (0.065, 0.457) |  |
| Emotional barriers | | |  | |  |  |  |  |  |  |  |  | |  |  |  |  |  |  |  |  | |  |  |  |  |  |  | |  |  |  |
|  | “Fear of positive result” | | -0.200** (-0.342, -0.058) | | -0.152* (-0.269, -0.034) | 0.154* (0.028, 0.281) | 0.035 (-0.129, 0.199) | 0.179 (-0.021, 0.379) | 0.006 (-0.039, 0.050) | -0.141 (-0.614, 0.331) | -0.231* (-0.430, -0.032) | 0.002 (-0.008, 0.011) | | -0.007 (-0.029, 0.015) | 0.057 (-0.150, 0.264) | 0.003 (-0.183, 0.189) | 0.028 (-0.110, 0.165) | -0.001 (-0.160, 0.158) | 0.151 (-0.012, 0.314) | 0.074 (-0.146, 0.294) | 0.368*** (0.191, 0.545) | | 0.103 (-0.094, 0.300) | 0.148 (-0.018, 0.313) | 0.122 (-0.210, 0.454) | -0.117 (-0.440, 0.206) | 0.058 (-0.069, 0.186) | 0.219** (0.077, 0.362) | | 0.459*** (0.262, 0.655) | 0.278** (0.081, 0.474) |  |
|  | “Embarrassment” | | -0.225** (-0.364, -0.086) | | -0.067 (-0.182, 0.048) | 0.225*** (0.101, 0.349) | 0.149 (-0.011, 0.309) | 0.299** (0.103, 0.495) | 0.003 (-0.041, 0.046) | -0.378 (-0.840, 0.084) | -0.144 (-0.338, 0.051) | -0.002 (-0.012, 0.007) | | -0.011 (-0.032, 0.011) | -0.122 (-0.324, 0.080) | -0.068 (-0.250, 0.114) | -0.061 (-0.196, 0.073) | -0.203* (-0.358, -0.047) | 0.021 (-0.138, 0.181) | 0.008 (-0.207, 0.223) | 0.115 (-0.058, 0.288) | | 0.065 (-0.127, 0.258) | 0.017 (-0.145, 0.179) | -0.078 (-0.402, 0.246) | 0.262 (-0.054, 0.577) | 0.004 (-0.121, 0.128) | 0.018 (-0.121, 0.157) | | 0.180 (-0.012, 0.372) | 0.032 (-0.160, 0.223) |  |
|  | “Fear of pain” | | -0.154* (-0.298, -0.010) | | 0.004 (-0.115, 0.123) | 0.205** (0.077, 0.334) | 0.204* (0.037, 0.370) | 0.378*** (0.175, 0.582) | -0.019 (-0.065, 0.026) | -0.361 (-0.841, 0.118) | -0.072 (-0.274, 0.130) | -0.005 (-0.015, 0.005) | | 0.010 (-0.013, 0.032) | 0.064 (-0.146, 0.274) | -0.016 (-0.205, 0.173) | -0.002 (-0.142, 0.137) | -0.189* (-0.350, -0.027) | 0.057 (-0.108, 0.223) | -0.005 (-0.228, 0.219) | -0.038 (-0.217, 0.142) | | -0.054 (-0.253, 0.146) | -0.056 (-0.224, 0.112) | -0.067 (-0.404, 0.270) | 0.264 (-0.063, 0.592) | -0.068 (-0.198, 0.062) | 0.022 (-0.123, 0.167) | | 0.186 (-0.013, 0.385) | 0.033 (-0.166, 0.232) |  |
|  | “Fear of radiation” | | -0.177** (-0.298, -0.056) | | 0.020 (-0.080, 0.120) | 0.181** (0.073, 0.289) | 0.099 (-0.041, 0.238) | 0.146 (-0.025, 0.317) | 0.011 (-0.027, 0.049) | -0.354 (-0.757, 0.048) | -0.099 (-0.269, 0.071) | 0.003 (-0.005, 0.012) | | -0.010 (-0.029, 0.009) | 0.144 (-0.033, 0.320) | 0.078 (-0.081, 0.236) | 0.032 (-0.085, 0.150) | -0.102 (-0.237, 0.034) | 0.093 (-0.046, 0.232) | -0.088 (-0.275, 0.100) | -0.028 (-0.179, 0.123) | | -0.084 (-0.252, 0.083) | -0.099 (-0.240, 0.042) | -0.134 (-0.417, 0.149) | 0.017 (-0.258, 0.293) | 0.027 (-0.081, 0.136) | 0.035 (-0.086, 0.157) | | 0.162 (-0.005, 0.330) | -0.026 (-0.194, 0.141) |  |
| Knowledge barriers | | |  | |  |  |  |  |  |  |  |  | |  |  |  |  |  |  |  |  | |  |  |  |  |  |  | |  |  |  |
|  | “No need to screen because of good health” | | -0.017 (-0.151, 0.116) | | 0.036 (-0.075, 0.146) | 0.063 (-0.056, 0.183) | 0.049 (-0.105, 0.203) | 0.089 (-0.100, 0.278) | 0.005 (-0.037, 0.047) | -0.132 (-0.577, 0.313) | -0.081 (-0.269, 0.106) | 0.003 (-0.007, 0.012) | | -0.009 (-0.030, 0.012) | 0.044 (-0.151, 0.239) | -0.008 (-0.184, 0.167) | 0.068 (-0.061, 0.198) | -0.124 (-0.274, 0.026) | 0.036 (-0.117, 0.190) | 0.027 (-0.180, 0.234) | 0.107 (-0.059, 0.274) | | -0.020 (-0.206, 0.165) | -0.077 (-0.233, 0.079) | 0.130 (-0.182, 0.442) | -0.149 (-0.453, 0.156) | 0.002 (-0.118, 0.122) | 0.032 (-0.102, 0.167) | | 0.022 (-0.163, 0.207) | -0.132 (-0.317, 0.053) |  |
|  | “No recommendation from my doctor” | | -0.027 (-0.169, 0.115) | | 0.045 (-0.073, 0.163) | -0.008 (-0.135, 0.119) | 0.054 (-0.110, 0.219) | -0.145 (-0.346, 0.056) | 0.000 (-0.045, 0.045) | -0.291 (-0.765, 0.183) | 0.055 (-0.145, 0.254) | 0.001 (-0.009, 0.011) | | -0.009 (-0.032, 0.013) | 0.185 (-0.023, 0.392) | 0.078 (-0.108, 0.265) | 0.079 (-0.059, 0.218) | -0.196* (-0.356, -0.037) | -0.006 (-0.170, 0.157) | 0.203 (-0.018, 0.423) | -0.104 (-0.281, 0.074) | | 0.013 (-0.184, 0.211) | -0.235** (-0.401, -0.068) | 0.203 (-0.130, 0.536) | -0.229 (-0.553, 0.095) | -0.086 (-0.215, 0.042) | 0.045 (-0.098, 0.188) | | -0.022 (-0.219, 0.175) | -0.040 (-0.237, 0.157) |  |
|  | “Lack of knowledge on service location” | | -0.475*** (-0.615, -0.335) | | -0.206*** (-0.322, -0.090) | 0.115 (-0.010, 0.240) | 0.046 (-0.115, 0.208) | 0.038 (-0.160, 0.236) | -0.029 (-0.074, 0.015) | -0.193 (-0.659, 0.273) | 0.097 (-0.099, 0.294) | 0.001 (-0.008, 0.011) | | -0.020 (-0.042, 0.001) | -0.072 (-0.276, 0.132) | -0.234* (-0.418, -0.050) | -0.071 (-0.206, 0.065) | 0.001 (-0.155, 0.158) | 0.075 (-0.086, 0.236) | 0.071 (-0.146, 0.288) | 0.086 (-0.089, 0.261) | | 0.040 (-0.154, 0.235) | -0.026 (-0.190, 0.137) | 0.162 (-0.165, 0.490) | -0.181 (-0.500, 0.138) | 0.056 (-0.070, 0.182) | 0.151* (0.010, 0.291) | | 0.308** (0.114, 0.501) | 0.102 (-0.092, 0.296) |  |
|  | “Lack of knowledge on MMG” | | -0.360*** (-0.492, -0.228) | | -0.113* (-0.222, -0.003) | 0.189** (0.071, 0.307) | 0.192* (0.040, 0.345) | 0.325*** (0.139, 0.512) | -0.009 (-0.051, 0.033) | -0.189 (-0.629, 0.252) | -0.026 (-0.211, 0.160) | -0.001 (-0.010, 0.008) | | -0.002 (-0.023, 0.018) | -0.138 (-0.331, 0.055) | -0.119 (-0.293, 0.054) | -0.102 (-0.230, 0.026) | -0.131 (-0.280, 0.017) | 0.057 (-0.095, 0.209) | -0.069 (-0.274, 0.136) | 0.083 (-0.082, 0.248) | | -0.025 (-0.208, 0.159) | -0.096 (-0.251, 0.058) | -0.081 (-0.390, 0.228) | 0.101 (-0.200, 0.402) | 0.112 (-0.007, 0.231) | 0.088 (-0.045, 0.221) | | 0.131 (-0.052, 0.314) | 0.106 (-0.077, 0.290) |  |
| Cues to action | | |  | |  |  |  |  |  |  |  |  | |  |  |  |  |  |  |  |  | |  |  |  |  |  |  | |  |  |  |
|  | “One-stop multiple cancer screening service” | | 0.053 (-0.064, 0.170) | | 0.035 (-0.062, 0.132) | -0.053 (-0.157, 0.051) | -0.099 (-0.234, 0.035) | -0.241** (-0.406, -0.076) | 0.007 (-0.030, 0.043) | 0.347 (-0.042, 0.735) | -0.036 (-0.200, 0.128) | 0.008* (0.000, 0.016) | | -0.019* (-0.037, -0.001) | 0.143 (-0.027, 0.313) | -0.085 (-0.238, 0.068) | -0.006 (-0.119, 0.108) | -0.067 (-0.198, 0.064) | -0.004 (-0.138, 0.130) | -0.193* (-0.374, -0.012) | 0.121 (-0.025, 0.266) | | 0.118 (-0.044, 0.280) | 0.143* (0.006, 0.279) | 0.067 (-0.206, 0.340) | -0.108 (-0.373, 0.158) | 0.066 (-0.039, 0.171) | 0.009 (-0.108, 0.126) | | -0.001 (-0.163, 0.160) | 0.048 (-0.114, 0.209) |  |
|  | “Fear of having BC” | | -0.079 (-0.200, 0.041) | | -0.014 (-0.114, 0.086) | 0.002 (-0.106, 0.109) | -0.019 (-0.159, 0.120) | -0.139 (-0.310, 0.031) | -0.013 (-0.051, 0.025) | 0.340 (-0.062, 0.742) | 0.051 (-0.118, 0.221) | 0.000 (-0.008, 0.009) | | 0.005 (-0.014, 0.024) | -0.083 (-0.259, 0.093) | -0.210** (-0.369, -0.052) | -0.111 (-0.228, 0.006) | -0.016 (-0.151, 0.119) | -0.021 (-0.160, 0.117) | -0.040 (-0.227, 0.147) | 0.217** (0.067, 0.367) | | 0.146 (-0.021, 0.314) | 0.087 (-0.054, 0.228) | 0.046 (-0.236, 0.328) | -0.022 (-0.297, 0.252) | 0.042 (-0.066, 0.151) | 0.011 (-0.111, 0.132) | | 0.053 (-0.114, 0.220) | 0.212* (0.045, 0.379) |  |
|  | “Healthcare professional recommendation” | | -0.059 (-0.172, 0.053) | | 0.065 (-0.029, 0.158) | 0.001 (-0.100, 0.101) | 0.015 (-0.115, 0.145) | -0.068 (-0.228, 0.091) | 0.009 (-0.026, 0.045) | 0.252 (-0.123, 0.627) | 0.151 (-0.008, 0.309) | 0.002 (-0.005, 0.010) | | -0.007 (-0.025, 0.010) | -0.139 (-0.303, 0.026) | -0.318*** (-0.466, -0.170) | -0.169** (-0.279, -0.060) | 0.018 (-0.108, 0.145) | -0.066 (-0.196, 0.064) | -0.233** (-0.407, -0.058) | 0.098 (-0.043, 0.238) | | 0.000 (-0.157, 0.156) | 0.045 (-0.087, 0.177) | 0.114 (-0.149, 0.378) | 0.095 (-0.162, 0.351) | 0.034 (-0.068, 0.135) | 0.035 (-0.079, 0.148) | | -0.017 (-0.173, 0.138) | 0.120 (-0.035, 0.276) |  |
|  | “Relative/friend recommendation” | | 0.072 (-0.042, 0.185) | | 0.037 (-0.056, 0.131) | 0.028 (-0.074, 0.129) | 0.022 (-0.109, 0.153) | -0.110 (-0.270, 0.051) | 0.012 (-0.024, 0.047) | 0.229 (-0.149, 0.607) | -0.001 (-0.160, 0.158) | -0.001 (-0.009, 0.007) | | 0.006 (-0.012, 0.024) | -0.250** (-0.415, -0.084) | -0.273*** (-0.422, -0.125) | -0.157** (-0.267, -0.046) | -0.029 (-0.156, 0.099) | 0.072 (-0.058, 0.202) | -0.058 (-0.234, 0.118) | 0.071 (-0.070, 0.213) | | 0.055 (-0.103, 0.212) | 0.092 (-0.041, 0.224) | -0.013 (-0.278, 0.253) | 0.050 (-0.209, 0.308) | 0.071 (-0.031, 0.174) | -0.002 (-0.116, 0.112) | | -0.029 (-0.186, 0.128) | 0.154 (-0.003, 0.311) |  |
|  | “Media information” | | 0.113 (-0.010, 0.236) | | 0.231*** (0.129, 0.332) | 0.029 (-0.080, 0.139) | 0.096 (-0.046, 0.238) | -0.039 (-0.213, 0.135) | -0.027 (-0.066, 0.011) | 0.168 (-0.242, 0.578) | 0.170 (-0.003, 0.343) | 0.006 (-0.002, 0.015) | | -0.007 (-0.026, 0.012) | -0.206* (-0.385, -0.027) | -0.373*** (-0.534, -0.211) | -0.160** (-0.279, -0.040) | -0.052 (-0.190, 0.086) | -0.102 (-0.244, 0.039) | -0.121 (-0.311, 0.070) | 0.188* (0.035, 0.342) | | 0.085 (-0.086, 0.255) | 0.082 (-0.061, 0.226) | 0.120 (-0.168, 0.407) | 0.279 (-0.001, 0.559) | -0.101 (-0.211, 0.010) | 0.013 (-0.111, 0.136) | | 0.036 (-0.134, 0.206) | 0.131 (-0.039, 0.302) |  |
|  | “Free-of-charge service” | | -0.088 (-0.212, 0.037) | | 0.037 (-0.066, 0.141) | -0.015 (-0.127, 0.096) | 0.070 (-0.074, 0.214) | 0.031 (-0.146, 0.207) | -0.001 (-0.040, 0.039) | 0.390 (-0.026, 0.806) | 0.056 (-0.119, 0.232) | -0.002 (-0.010, 0.007) | | 0.000 (-0.019, 0.020) | 0.097 (-0.085, 0.280) | 0.007 (-0.156, 0.171) | 0.053 (-0.068, 0.174) | -0.049 (-0.189, 0.091) | -0.009 (-0.152, 0.135) | -0.182 (-0.375, 0.012) | 0.051 (-0.104, 0.207) | | 0.106 (-0.067, 0.279) | 0.043 (-0.103, 0.188) | -0.021 (-0.313, 0.271) | 0.066 (-0.218, 0.351) | 0.080 (-0.033, 0.192) | 0.012 (-0.114, 0.137) | | -0.019 (-0.192, 0.154) | 0.100 (-0.073, 0.273) |  |
|  | “Benefits of BC screening” | | 0.017 (-0.097, 0.131) | | 0.088 (-0.006, 0.183) | -0.016 (-0.118, 0.085) | -0.061 (-0.193, 0.070) | -0.160 (-0.321, 0.001) | 0.011 (-0.025, 0.047) | 0.548** (0.169, 0.928) | 0.049 (-0.111, 0.209) | 0.002 (-0.006, 0.009) | | -0.003 (-0.021, 0.015) | 0.112 (-0.054, 0.278) | -0.059 (-0.208, 0.091) | 0.005 (-0.106, 0.115) | -0.004 (-0.132, 0.124) | 0.057 (-0.074, 0.188) | -0.170 (-0.347, 0.006) | 0.004 (-0.138, 0.146) | | 0.115 (-0.043, 0.273) | 0.095 (-0.038, 0.228) | 0.079 (-0.188, 0.346) | -0.091 (-0.350, 0.168) | 0.055 (-0.047, 0.158) | -0.024 (-0.139, 0.090) | | -0.007 (-0.165, 0.150) | 0.030 (-0.128, 0.187) |  |
| Type | Outcome | | OR | | OR | OR | OR | OR | OR | OR | OR | OR | | OR | OR | OR | OR | OR | OR | OR | OR | | OR | OR | OR | OR | OR | OR | | OR | OR |  |
| Risk concordance | | |  | |  |  |  |  |  |  |  |  | |  |  |  |  |  |  |  |  | |  |  |  |  |  |  | |  |  |  |
|  | Concordant BC risk perception | | 0.572* (0.341, 0.956) | | 1.034 (0.648, 1.640) | 1.019 (0.627, 1.658) | 1.729 (0.921, 3.297) | 1.161 (0.565, 2.414) | 0.949 (0.807, 1.120) | 0.256 (0.058, 1.331) | 0.302*** (0.157, 0.584) | 0.988 (0.952, 1.025) | | 1.044 (0.961, 1.135) | 0.291** (0.134, 0.619) | 0.372** (0.182, 0.744) | 0.737 (0.408, 1.298) | 0.680 (0.379, 1.250) | 1.457 (0.783, 2.854) | 1.314 (0.602, 3.087) | 0.875 (0.463, 1.682) | | 0.839 (0.405, 1.768) | 0.942 (0.510, 1.760) | 1.189 (0.367, 4.451) | 0.479 (0.155, 1.689) | 1.188 (0.751, 1.862) | 0.924 (0.554, 1.550) | | 2.017 (0.916, 4.734) | 1.319 (0.618, 2.889) |  |

This table shows the full results of Table 3. BC; breast cancer. MMG; mammography. Coef.; coefficients. OR; odds ratio. 95% confidence intervals are in parenthesis. * *p* <0.05; ** *p* <0.01; *** *p* <0.001. Multiple linear regression was used to estimate the coefficients, except for risk concordance whose coefficients are odds ratios estimated by logistic regression. 120 participants were excluded from the model due to missing data on household income (N=120) and employment status (N=9). The study was conducted in Hong Kong from December 2020 to April 2022.

Table S2. Associations between health literacy score (HLS-SF12) and covariates (N=701)

| Independent Variable | Category | Univariable | Multivariable |
| --- | --- | --- | --- |
|  |  | Coefficient | Coefficient |
| Age | 50-54 | Ref | Ref |
|  | 55-59 | -0.317 (-1.539, 0.905) | 0.089 (-1.119, 1.296) |
|  | 60-64 | -2.052** (-3.404, -0.699) | -0.656 (-2.218, 0.907) |
|  | 65+ | -3.071*** (-4.653, -1.489) | -1.552 (-3.463, 0.359) |
| Number of chronic diseases |  | -0.464* (-0.899, -0.028) | -0.167 (-0.594, 0.259) |
| History of other cancers |  | 2.011 (-2.670, 6.692) | 0.641 (-3.866, 5.147) |
| Family history of breast cancer |  | -0.565 (-2.554, 1.423) | -0.300 (-2.201, 1.600) |
| Waist circumference |  | -0.059* (-0.118, -0.001) | -0.041 (-0.134, 0.052) |
| BMI |  | -0.095 (-0.228, 0.037) | 0.012 (-0.199, 0.223) |
| Education | College/university | Ref | Ref |
|  | Primary school | -6.773*** (-8.354, -5.192) | -5.701*** (-7.619, -3.783) |
|  | Secondary school 1-3 | -3.630*** (-5.145, -2.115) | -2.870** (-4.612, -1.128) |
|  | Secondary school 4-7 | -1.924** (-3.118, -0.730) | -1.484* (-2.781, -0.187) |
| Marital status | Married/cohabitating | Ref | Ref |
|  | Unmarried | -0.003 (-1.519, 1.513) | -0.973 (-2.489, 0.543) |
|  | Separated/divorced | -0.955 (-2.543, 0.633) | -0.795 (-2.352, 0.761) |
|  | Widowed | -3.125** (-5.279, -0.971) | -1.417 (-3.513, 0.679) |
| Employment status | Full-time | Ref | Ref |
|  | Part-time | -1.761* (-3.266, -0.257) | 0.094 (-1.595, 1.783) |
|  | Retired | -1.322 (-2.838, 0.194) | 1.312 (-0.563, 3.188) |
|  | Housewife | -0.107 (-1.381, 1.167) | 2.560** (0.993, 4.128) |
|  | Unemployed | -2.515 (-5.603, 0.573) | -0.670 (-3.837, 2.498) |
|  | Self-employed | 3.577* (0.409, 6.744) | 3.841* (0.767, 6.916) |
| Born in Hong Kong |  | 2.492*** (1.318, 3.665) | 1.197 (-0.018, 2.412) |
| Monthly income | 10,000 or below | Ref | Ref |
|  | 10,001-20,000 | 1.461* (0.304, 2.617) | 0.750 (-0.609, 2.109) |
|  | 20,001-30,000 | 2.406** (0.823, 3.989) | 0.641 (-1.231, 2.514) |
|  | 30,001 or above | 3.682*** (2.212, 5.152) | 1.519 (-0.355, 3.393) |

95% confidence intervals are in parenthesis. * *p* <0.05; ** *p* <0.01; *** *p* <0.001. Univariable and multivariable linear regression was used to estimate the coefficients. 120 participants were excluded from the model due to missing data on household income (N=120) and employment status (N=9).
